# The Oxidative Damage and Inflammation Mechanisms in GERD-Induced Barrett’s Esophagus

**DOI:** 10.3389/fcell.2022.885537

**Published:** 2022-05-26

**Authors:** Deqiang Han, Chao Zhang

**Affiliations:** ^1^ Department of General Surgery, National Clinical Research Center for Geriatric Diseases, Xuanwu Hospital of Capital Medical University, Beijing, China; ^2^ Cell Therapy Center, Beijing Institute of Geriatrics, Xuanwu Hospital Capital Medical University, National Clinical Research Center for Geriatric Diseases, Beijing, China

**Keywords:** Barrett’s esophagus, DNA damage, transdifferentiation, polyADP-ribose polymerase 1, NF-kappa B

## Abstract

Barrett’s esophagus is a major complication of gastro-esophageal reflux disease and an important precursor lesion for the development of Barrett’s metaplasia and esophageal adenocarcinoma. However, the cellular and molecular mechanisms of Barrett’s metaplasia remain unclear. Inflammation-associated oxidative DNA damage could contribute to Barrett’s esophagus. It has been demonstrated that poly(ADP-ribose) polymerases (PARPs)-associated with ADP-ribosylation plays an important role in DNA damage and inflammatory response. A previous study indicated that there is inflammatory infiltration and oxidative DNA damage in the lower esophagus due to acid/bile reflux, and gastric acid could induce DNA damage in culture esophageal cells. This review will discuss the mechanisms of Barrett’s metaplasia and adenocarcinoma underlying oxidative DNA damage in gastro-esophageal reflux disease patients based on recent clinical and basic findings.

## Introduction

Barrett’s esophagus (BE) most commonly arises from gastro-esophageal reflux disease (GERD), which is defined as the retrograde flow of gastric and (or) duodenal contents into the esophagus, inducing discomfort symptoms and (or) esophageal mucosal pathological lesions. The incidence rate of GERD varies from 10 to 20% in the Eastern and Western countries (El- Serag et al., 2014; Amadi et al., 2017; Maslyonkina et al., 2021; Mittal et al., 2021). Indeed, it is one of the most prevalent gastrointestinal functional disorders worldwide. Heartburn and regurgitation are the typical symptoms of GERD and the number of atypical manifests is estimated over 100, including non-cardiac chest pain, bronchial-pulmonary or ear, nose, and throat symptoms, and dental erosion. The understanding of the cellular and molecular mechanisms by which this metaplastic transformation occurs remains limited. Histological proof of BE is currently considered objective evidence of GERD, which is a common pre-malignant condition characterized by the replacement of the normal squamous epithelium by a metaplastic columnar-lined epithelium extending to the gastro-oesophageal junction (Barrett, 1950; McDonald et al., 2015). GERD is a chronic inflammation of the esophagus stimulated by repeated acid/bile acids. Chronic inflammation is likely to carry an increased risk of cancer via oxidative damage pathways (Farinati et al., 2010). Therefore, both GERD-induced reactive oxygen species (ROS) accumulation and chronic inflammatory infiltration are associated with BE formation. This review mainly focuses on the oxidative damage, inflammation mechanisms, and the reparative response in GERD-induced BE.

## Inflammation Involved in GERD-Induced Barrett’s Esophagus

GERD is a chronic inflammation of the esophagus stimulated by repeated acid/bile acids. Thus, esophagitis is the major pathological manifestation for patients with GERD and BE (Vaezi and Richter, 1996; Deshpande et al., 2021). Furthermore, chronic inflammation is likely to carry an increased risk of cancer via oxidative damage pathways (Farinati et al., 2010). Metaplasia is a pathological condition that commonly occurs in the presence of chronic inflammation (Kumar AKA et al., 2007), including typical Barrett’s metaplasia (Colleypriest et al., 2009). Therefore, there is a strong association between GERD-induced reactive oxygen species (ROS) accumulation, chronic inflammatory infiltration, and BE formation. Some cell fate and development-related genes transcriptionally change in the conditions of such chronic inflammation, including BMP4, PTGS2, SHH, CDX1, CDX2, Notch, and SOX9 (Jiang et al., 2017; Peters et al., 2019).

The reflux-induced epithelial injury could be repaired by squamous cell regeneration and differentiated columnar epithelium in the distal esophagus. It is reviewed that different types of cells have been proposed to develop intestinal metaplasia during GERD-induced BE by direct or indirect trans-differentiation. Many signaling pathways may also be involved in this process (Peters et al., 2019). Columnar epithelium may be an intermediate stage in the formation of specialized intestinal metaplasia that pSMAD/CDX2 interaction is essential for the switch toward an intestinal phenotype (Mari et al., 2014). An inflammatory environment induced by damage leads to increased sonic hedgehog signaling and decreased Notch signaling mediated by PGE2, NF-κB, TNF, and other molecules. In addition, genetic variations are involved in BE. Variants of GSTP1 (such as rs1695A > G missense variant) are frequently linked to risks of infiltration and esophageal adenocarcinoma (EAC) due to the reduction of antioxidant enzymatic activity (Peng et al., 2021). Therefore, the detailed molecular mechanism of oxidative damage and inflammation involved in GERD-induced BE should be further explored.

## Role of p63 in Barrett’s Esophagus

BE may arise and develop from various stem cells, including residual embryonic stem cells, submucosal gland stem cells, gastric cardia stem cells, gastro-oesophageal junction, or basal squamous progenitor cells (Badgery et al., 2020). BE is defined as the replacement of the normal squamous epithelium by a metaplastic columnar-lined epithelium. Abnormal differentiation of multipotential stem cells into columnar-lined epithelium was considered one of the potential mechanisms (Tosh and Slack, 2002). p63, the p53 gene family member, has been termed as the master regulator of epithelial cells that determines the differentiation of progenitor cells into squamous epithelium cells. In p63^−/−^ mice, the stratified squamous epithelium fails to form, while the esophagus is lined by simple columnar epithelium (Daniely et al., 2004; Koster et al., 2004). Consistently, BE lacks the staining of p63 (Daniely et al., 2004; von Holzen and Enders, 2012). Thus, Barrett’s stem cells may not be derived from the p63^+^ embryonic esophageal progenitor cells and the adult squamous esophageal stem cells. The other possibility would be that p63 is downregulated in originally p63^+^ adult squamous esophageal stem cells in BE. Indeed, downregulation of p63 was observed upon exposure to bile salts and acid in normal and cancer esophageal cells in culture (Roman et al., 2007). Thus, it is more likely that p63^+^ adult squamous esophageal stem cells lost p63 expression in BE due to the repeated acid/bile acid stimulation in GERD patients. Molecular mechanisms for p63 downregulation in BE need to be further investigated.

## DNA Damage Repair in GERD-Induced Barrett’s Esophagus

The pathological mechanism of GERD is relatively clear, including the lower esophageal sphincter and cardia relaxation, lower esophageal sphincter pressure, and (or) esophageal insufficiency, esophageal hiatal hernia, leading to gastric acid, pepsin, and bile reflux into the esophagus. Bile salts or hydrochloric acid treatment could increase the levels of ROS, inducing an increase in the levels of 8-hydroxydeoxyguanosine (8-OH-dG) and p-H2AX which are markers of oxidative DNA damage and DNA double-strand breaks, respectively (Zhang et al., 2009; Dvorak et al., 2007). It is well established that oxidative DNA damage is usually induced by ROS which is primarily generated from normal intracellular metabolism in mitochondria and peroxisomes (Cadet et al., 2010). Increased studies from clinical biopsies have shown that oxidative stress exists in the GERD model as well as BE (Dvorak et al., 2007; Räsänen et al., 2007). Chronic exposures to high levels of ROS from overwhelming reflux and the deteriorative ability of bolus clearance in the esophagus, these excessive active free radicals to attack genomic DNA and consequently induce various types of DNA lesions. These lesions, including DNA single-stand breaks and double-strand breaks, may lead to genomic instability and various diseases (Olinski et al., 2002; Sedelnikova et al., 2010). Recent studies reported that oxidative DNA damage exists in Barrett’s mucosa, and the magnitude of damage is beyond the repair capacity of a cell (Cardin et al., 2013). Since GERD patients developed BE or EAC with an approximately 6–8 fold increased risk than normal people, BE patients carry an increased risk of EAC varying between 30–125 times that of the general population (Altorki et al., 1997). Both CD133 and 8-OH-dG formation were detected at the apical surface of columnar epithelial cells of biopsy specimens of patients with BE and BE adenocarcinoma with significantly higher expression levels. This study indicated that oxidative and nitrative DNA damage and CD133 localization would contribute to BE-derived carcinogenesis (Thanan et al., 2016). Corresponding to the repair of oxidative DNA damage, apurinic/apyrimidinic endonuclease 1 (APE1), one of the key enzymes generated by ROS, is frequently overexpressed in EAC. Moreover, Barrett’s and EAC cells could be protected against oxidative DNA damage by regulating JNK and p38 kinases (Hong et al., 2016; Peng et al., 2021). In this regard, the relationship between oxidative DNA damage and BE progress should be further explored. Next, we will especially discuss the role of the ROS/PARP-1/NF-κB pathway in the formation of BE and Barrett’s adenocarcinoma.

## The Role of PARP-1 in Barrett’s Esophagus

Oxidative stress triggers DNA strand breakage in BE, leading to the activation of the nuclear enzyme poly(ADP-ribose) polymerase (PARPs) which catalyze poly(ADP-ribose)relation (PARylation) at the sites of damage (Liu and Yu, 2015). These enzymes use NAD^+^ as the substrate and the negatively charged ADP-ribose (ADP) group is covalently added to the target proteins. The most common target sites for PARylation are the side chains of arginine, aspartic acid, and glutamic acid residues. After catalyzing the addition of the first ADPr onto the target proteins, other ADPrs can be covalently polymerized onto the first ADPr leading to the formation of both linear and branched polymers, called poly(ADP-ribose) (PAR) (Schreiber et al., 2006). Among the PARP family, PARP-1 is the prototypical and most abundant nuclear-expressed PARPs, which can PARylate various target proteins, including histones, DNA polymerases, DNA ligase, and PARP-1 itself. The PAR chains generated by PARP-1 form various regulatory complexes during DNA damage response and metabolism (Kim et al., 2005; Malanga and Althaus, 2005; Schreiber et al., 2006). PARP-1 can be excessively activated in situations where oxidative DNA damage is beyond the repair capacity of PARP-1. These conditions lead to excessive consumption of NAD^+^. Since NAD^+^ synthesis requires ATP molecules, the reduction of cellular NAD^+^ and ATP levels leads to the collapse of cellular metabolism and, consequently, cell death (Schreiber et al., 2006). Thus, the PARP-1 upregulation may present a double-edged sword in the process of DNA damage response.

Recently, the role of PARP-1-dependent DNA damage response in the formation of BE and the pathological process of GERD-induced esophageal cancer is very limited. Our preliminary results show that PARP1 overexpression is probably taken as a resistance factor of BE epithelial cells to H_2_O_2_ or bile acid-induced oxidative damage and cell death. PARP1 also positively regulates the viability of esophageal epithelial cells, which reveals a potential candidate for a therapeutic strategy for BE (Zhang et al., 2018). PARP-1 is also known to be a co-activator of NF-κB, playing a key role in pro-inflammation by contributing to inflammatory processes through the regulation of transcription factors (Hassa and Hottiger, 1999; Liu et al., 2012). NF-κB was one of the first mediators of inflammation to be identified as a target for PARP-1 mediated PARylation (Aguilar-Quesada et al., 2007). In PARP^−/−^ mice and cell lines, NF-κB activity is severely compromised in absence of activation by upstream PARP-1 (Oliver et al., 1999), and in an oxazolone-induced contact hypersensitivity model, PARP-1 inhibition reduces the extent of inflammation by modulating oxidative stress and impairing the activation of NF-κB (Brunyánszki et al., 2010). PARP-1 may serve as a negative regulator of p63 by activating NF-κB in Barrett’s cell. Hence, oxidative stress-induced high PARP-1 activity in the BE-related stem cells may downregulate p63.

## Future Perspectives

**FIGURE 1:**
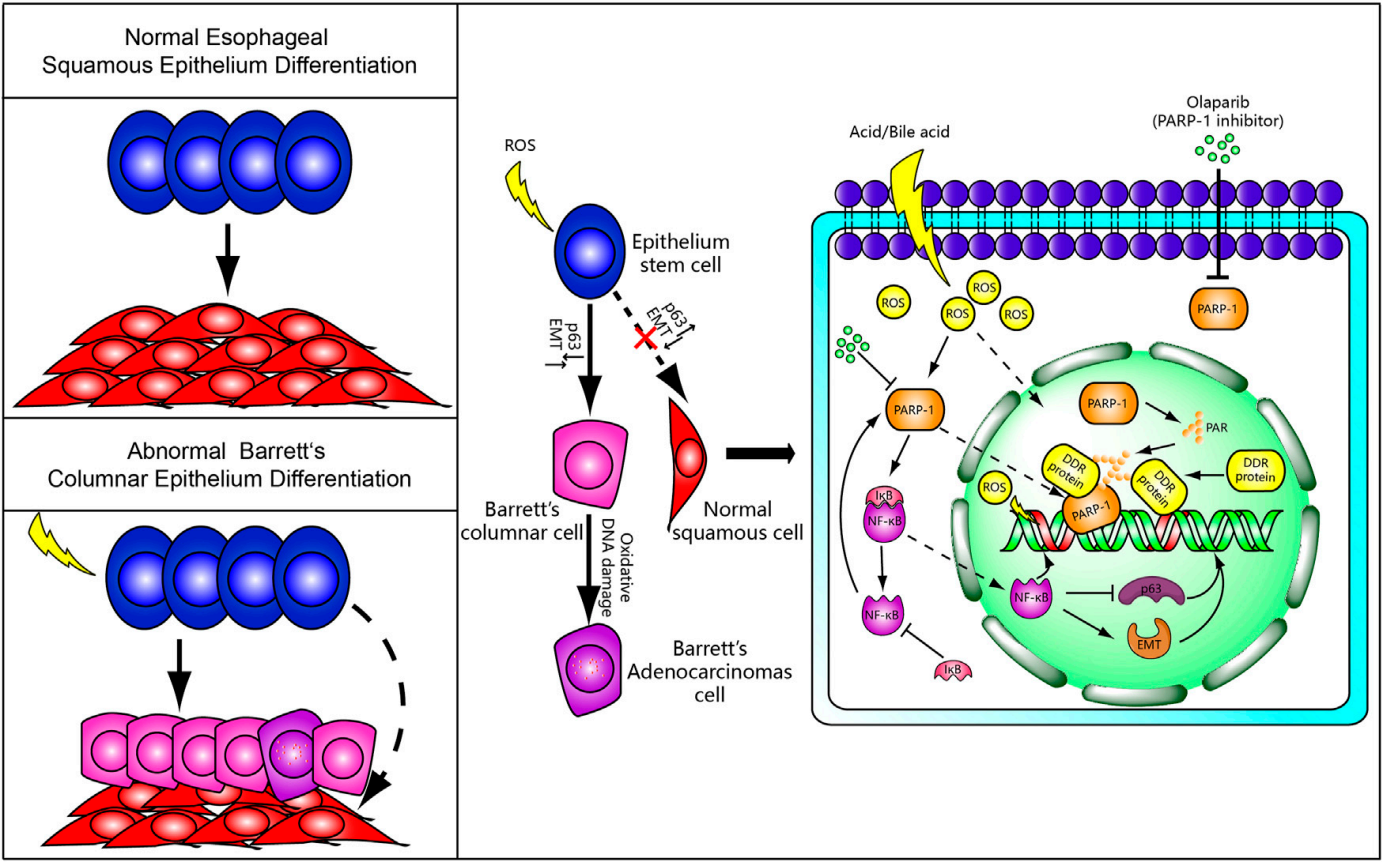
A putative mechanism for Barrett’s metaplasia and adenocarcinoma. Normally, epithelial stem cells differentiate into squamous epithelium cells. However, Barrett’s columnar epithelium cells and adenocarcinoma cells replace the normal squamous cells by abnormal differentiation under chronic reflux-induced oxidative damage and inflammation. We speculate that PARP-1/NF-κB signaling and ADP-ribosylation-dependent DNA damage response may be involved in the occurrence of BE and incomplete DNA repair possibly lead to Barrett’s adenocarcinoma. The PARP-1 inhibitor may serve as a molecular rescuer for BE formation.

The widely present evidence of oxidative DNA damage in BE from human tissue and cell models was recently reported (Dvorak et al., 2007; Peng et al., 2014; Hong et al., 2016). It is assumed that the development of BE is associated with oxidative DNA damage response. The long-term excessive acid/base-induced ROS stimulation in GERD may lead to activation of the PARP-1/NF-κB pathway with inflammatory infiltration of the epithelial stem cells. The inflammatory cells then tend to differentiate into Barrett’s esophageal epithelium (columnar epithelium) via transcription factor p63 and EMT. Whereas, DNA damage itself can lead to carcinogenesis with incomplete ADP-ribosylation-dependent DNA damage response. All these events can be associated with a heterogeneity of esophageal epithelial cells and tumor occurrence and development, eventually leading to EAC (Figure 1). This presumably suggests that antagonists of PARP-1/NF-κB might have beneficial effects on Barrett’s metaplasia in GERD patients. However, to the best of our knowledge, there has been no research on the effects of oxidative DNA damage-related agents on Barrett’s cell lines or animal models, which necessitates more studies.

We speculate that PARP-1/NF-κB signaling and ADP-ribosylation-dependent DNA damage response may be involved in the occurrence of BE and incomplete DNA repair due to low levels of ADP-ribosylation possibly lead to Barrett’s adenocarcinoma. However, there are still many open questions existing in the field that require further studies. 1) The degree of oxidative DNA damage, the level of PARP-1/NF-κB signaling, and NAD^+^ between esophageal stem cells, esophageal squamous cells, Barrett’s esophageal columnar cells, and adenocarcinoma cells by acid, bile acid, and oxidative stress treatment should be further investigated. 2) Adding extrinsic NF-κB or activating PARP-1/NF-κB signaling to detect the DNA damage repair and inflammatory response between PARP-1 knockout esophageal stem cell lines and wild-type cell lines with induced ROS. So what is the detailed mechanism among NF-κB/∆Np63/EMT in the development of BE and EAC. 3) What is the exact role of PARP-1 in esophageal stem cells, Barrett’s esophageal columnar cells, and adenocarcinoma cells.
